# Effects of protease supplementation on the ileal digestibility of amino acids for protein ingredients in broiler chickens

**DOI:** 10.1016/j.psj.2025.105605

**Published:** 2025-07-25

**Authors:** Jinsu Hong, Jorge Perez-Palencia, Rob Patterson, Crystal Levesque

**Affiliations:** a Department of Animal Science, South Dakota State University, Brookings, SD 57007, USA; b Department of Animal Science, University of Minnesota, Saint Paul, MN 55108, USA; c CBS Bio Platforms Inc., Calgary, AB T2C 0J7, Canada

**Keywords:** Canola meal, Corn dried distillers grains with solubles, Enzyme, High-protein DDGS, Soybean meal

## Abstract

This study aimed to evaluate the effects of dietary protease supplementation on the apparent ileal digestibility (**AID**) and standardized ileal digestibility (**SID**) of amino acids (**AA**) in five protein feedstuffs fed to broilers. A total of 504 one-day-old male broiler chickens (Cobb 500) were randomly assigned to 84 cages in Petersime battery cages (6 birds/cage). From days 0 to 14, all birds were fed a commercial starter diet. From day 14 onward, birds were randomly assigned to one of ten dietary treatments in a 5 × 2 factorial design (8 replicates/treatment), consisting of five protein sources, including soybean meal (**SBM**), expeller-extruded SBM (**ESBM**), canola meal (**CM**), corn dried distillers grains with solubles (**DDGS**), and high-protein DDGS (**HP-DDGS**), and two protease dosages (0 and 250 mg/kg). Experimental diets were provided from days 14 to 21. Additionally, four cages were fed N-free diets with or without protease. On day 21, ileal digesta samples were collected after euthanasia. Data were analyzed using two-way ANOVA with the MIXED procedure of SAS to evaluate the main effects of protein source, protease dosage, and their interactions. In the present study, there were interactive effects (*P* < 0.05) between ingredient and protease on AID and SID of His, Ile, Leu, Phe, Thr, Val, and nonessential AA. Specifically, dietary protease improved (*P* < 0.05) the AID and SID of these AA for SBM and HP-DDGS. However, broilers fed ESBM, CM and DDGS diets supplemented with protease had no improvement in the AID and SID of these AA. In conclusion, protease supplementation could enhance the SID of AA in SBM and HP-DDGS in broilers, but its effects were less pronounced in ESBM, CM and DDGS. The lack of improvement in AA digestibility for ESBM, CM and DDGS may be attributed to their physiochemical properties, including the complexity and composition of the protein-fiber matrix, and antinutritional factors compared to other protein sources.

## Introduction

Soybean meal (**SBM**) is the most widely used protein source in poultry diets due to its high protein content and favorable amino acid (**AA**) profile. Canola meal (**CM**) and corn protein sources, including corn distillers dried grains with solubles (**cDDGS**) and high-protein DDGS (**HP-DDGS**) are promising alternative protein sources due to their high protein content and availability, and are increasingly used for their cost-effectiveness and sustainability benefits. To further enhance nutrient availability from these protein sources, feed enzymes such as phytase, carbohydrases, or proteases are commonly added to poultry diets. Among these exogenous enzymes, proteases are widely used in monogastric diets. Their supplementation increases the rate of hydrolysis of intact proteins, improving AA availability and reducing N excretion (Lee et al., 2018; Park et al., 2020a).

Previous studies have reported that protease supplementation improved nutrient digestibility and growth performance in broilers (Ghazi et al., 2002; Park et al., 2020a). In addition, protease supplementation tended to reduce the effects of protein anti-nutritional compounds or anti-nutritional factors such as trypsin inhibitor, glycinin, or beta-conglycinin, which are present in certain protein sources such as SBM and can negatively affect growth performance in young monogastric animals (Wang et al., 2014; Erdaw et al., 2017a; Park et al., 2020b). Taken together, protease supplementation in diets for young broilers has the potential to improve the digestibility of different protein sources.

The effectiveness of protease in poultry diets can be variable and has been associated with the type of protease, dosage, feed ingredients used in formulation, and interactions with other enzymes (Cowieson and Roos, 2016; Lee et al., 2018). Many studies have investigated the effects of protease supplementation on growth performance and health of broilers fed low protein diets (Ding et al., 2016; Lee et al., 2018) or as part of an enzyme blend for fibrous feedstuffs (Swiatkiewicz et al., 2016). However, there is a need for digestibility studies that focus on the impact of exogenous protease on individual protein ingredients. For example, the effect of dietary protease on the ileal digestibility of individual AA differed between SBM and rapeseed meal (**RSM**) in broilers (Liu et al., 2024). Thus, a greater understanding of how protease supplementation impacts AA availability as influenced by protein sources in broilers will provide key insights for optimal utilization of proteases to enhance growth performance of chickens.

Based on this background, we hypothesized that dietary protease supplementation would have a greater effect on AA digestibility in broilers fed protein source with higher protein content and a less complex protein-fiber matrix. The objective of this study was to determine the effects of dietary protease supplementation on apparent ileal digestibility (**AID**) and standardized ileal digestibility (**SID**) of AA for commonly used protein ingredients in broiler chickens.

## Materials and methods

The experimental animal procedures were reviewed and approved by the Institutional Animal Care and Use Committee at South Dakota State University, Brookings, SD, USA (SDSU-IACUC #2111-072E).

### Experimental diets

Corn starch-based diets with five protein ingredients were used in this study. The protein ingredients including SBM, extruded soybean meal (**ESBM**), cDDGS, HP-DDGS, and CM were formulated as a sole protein source in the diets. The SBM was solvent-extracted SBM, which was sourced from South Dakota Soybean Processors (Volga, SD, USA), and the ESBM was sourced from Lester Feed and Grain (Lester, IA, USA). The cDDGS and HP-DDGS were provided by POET (Sioux falls, SD, USA). The CM was solvent-extracted CM, which was sourced from Cargill (West Fargo, ND, USA).

Nitrogen free diet was used to estimate basal ileal endogenous AA losses required to calculate the SID of AA. The experimental diets were formulated to have a similar crude protein (**CP**) content using analyzed CP values for SBM, cDDGS, and HP-DDGS, and published CP values from the NRC (1994) for ESBM and CM. The experimental diets were designed to meet or exceed NRC (1994) nutrient recommendations for broiler chickens (Table 1).

**Table 1:** Experimental diets formulation and calculated composition (as-fed basis).

Item^1^	SBM diet	ESBM diet	CM diet	DDGS diet	HP-DDGS diet	Nitrogen-free diet
Ingredient, %						
Corn starch	48.05	46.05	39.70	28.62	49.33	68.79
Sucrose/sugar	10.00	10.00	10.00	10.00	10.00	15.00
SBM	35.00	0.00	0.00	0.00	0.00	0.00
Extruded SBM	0.00	37.00	0.00	0.00	0.00	0.00
Canola meal	0.00	0.00	44.00	0.00	0.00	0.00
DDGS	0.00	0.00	0.00	55.00	0.00	0.00
HP-DDGS	0.00	0.00	0.00	0.00	33.00	0.00
Cellulose (sulkafloc)	0.00	0.00	0.00	0.00	0.00	5.00
Soybean oil	2.00	2.00	2.00	2.00	2.00	5.00
Limestone	1.65	1.70	1.20	2.43	1.88	1.68
Monocalcium phosphate	1.75	1.70	1.55	0.55	1.90	2.35
Poultry vitamin premix^2^	0.25	0.25	0.25	0.25	0.25	0.25
Poultry mineral premix^3^	0.25	0.25	0.25	0.25	0.25	0.25
Choline chloride	0.30	0.30	0.30	0.30	0.30	0.30
Salt	0.45	0.45	0.45	0.30	0.45	0.50
Potassium chloride	0.00	0.00	0.00	0.00	0.34	0.58
Titanium dioxide	0.30	0.30	0.30	0.30	0.30	0.30
Calculated nutrient composition						
Crude protein, %	16.58	16.64	16.63	16.81	16.66	0.23
Calcium, %	1.00	1.00	1.00	1.00	1.00	1.00
STTD P, %	0.45	0.45	0.45	0.45	0.45	0.45
Sodium, %	0.21	0.21	0.21	0.21	0.21	0.21
Gross energy, kcal/kg	4,040	4,103	4,109	4,132	4,111	3,996

1 SBM: solvent-extracted soybean meal, ESBM: extruded soybean meal, CM: solvent-extracted canola meal, DDGS: corn dried distillers grains with solubles, and HP-DDGS: high-protein corn dried distillers grains with solubles.

2 Provided the following per kilogram of diet: 10,000 IU vitamin A, 5,000 IU vitamin D_3_, 80 IU vitamin E, 3 mg thiamine, 9 mg riboflavin, 60 mg nicotinic acid, 15 mg pantothenic acid, 2 mg folic acid, 4 mg pyridoxine, and 0.15 mg biotin.

3 Provided the following per kilogram of diet: 100 mg manganese, 40 mg iron, 15 mg copper, 100 mg zinc, 1 mg iodine, and 0.35 mg selenium.

The protease product was supplemented at 250 mg protease/kg of diet and was provided by CBS Bio Platforms Inc. (ProSparity 250, Calgary, AB, Canada). The enzymes present in the preparation are derived from microbial fermentation of *Bacillus licheniformis* and *Bacillus subtilis* and is formulated to contain minimum protease activity of 45,000 HUT units/g. It should be noted that one HUT unit (hemoglobin units on a tyrosine basis) corresponds to the amount of enzyme that produces in one minute, under specified conditions, a hydrolysate whose absorbance at 275 nm is the same as that of a solution containing 0.275 microgram/ml of tyrosine in 0.006 N HCl. The enzyme preparation was added to the diets using part of dietary corn starch as a carrier, and it was included on top based on the manufacturer’s recommendations for poultry diets. The experimental diets were prepared in mash form and contained titanium dioxide at 0.3 % as an indigestible marker to determine the AA digestibility. The analyzed nutrient composition of the protein ingredients and diets were presented in Table 2 and 3, respectively.

**Table 2:** Analyzed nutrient composition of protein ingredients^1^ (as-fed basis).

Item	SBM	ESBM	CM	DDGS	HP-DDGS
Dry matter, %	89.52	94.50	90.10	90.43	92.34
Crude protein, %	47.22	40.80	42.14	28.82	53.45
Ether extract, %	1.28	8.45	2.67	6.48	4.50
Crude ash, %	6.14	5.51	4.71	3.79	3.14
Crude fiber, %	3.64	5.78	14.53	8.60	6.68
Essential amino acids, %					
Arg	3.33	2.94	2.55	1.38	2.39
His	1.24	1.08	1.14	0.83	1.43
le	2.24	1.98	1.71	1.12	2.44
Leu	3.63	3.15	2.96	3.26	6.75
Lys	3.08	2.71	2.39	1.05	1.93
Met	0.67	0.58	0.83	0.63	1.14
Phe	2.42	2.10	1.72	1.32	2.84
Thr	1.85	1.64	1.72	1.13	2.14
Trp	0.65	0.54	0.47	0.21	0.42
Val	2.26	1.98	2.15	1.42	2.92
Nonessential amino acids, %					
Ala	2.05	1.81	1.82	1.98	4.07
Asp	5.42	4.72	2.99	1.91	3.97
Cys	0.76	0.65	1.12	0.69	1.04
Glu	8.73	7.65	7.81	4.45	9.19
Gly	2.00	1.82	2.15	1.17	2.09
Pro	2.31	2.05	2.47	2.35	4.15
Ser	2.10	1.88	1.53	1.29	2.46
Tyr	1.66	1.42	1.15	1.07	2.08

1 SBM: solvent-extracted soybean meal, Ex-SBM: extruded soybean meal, CM: solvent-extracted canola meal, DDGS: corn dried distillers grains with solubles, and HP-DDGS: high-protein corn dried distillers grains with solubles.

**Table 3:** Analyzed composition of experimental diets^1^ (as-fed basis).

Item	SBM diet	ESBM diet	CM diet	DDGS diet	HP-DDGS diet	N-free diet
Analyzed nutrient composition						
Dry matter, %	91.25	92.89	91.29	92.65	93.04	92.80
Crude protein, %	16.93	16.22	17.50	16.19	17.04	0.32
Ether extract, %	1.23	2.18	1.97	3.74	2.01	2.46
Crude ash, %	6.14	5.90	5.82	6.10	4.90	4.75
Crude fiber, %	1.29	2.18	4.67	4.76	1.76	2.66
Gross energy, kcal/kg	3,902	4,030	3,911	4,120	4,053	4,111
Essential amino acids, %						
Arg	1.23	1.14	1.15	0.72	0.77	0.01
His	0.46	0.43	0.52	0.46	0.47	0.01
Ile	0.84	0.77	0.79	0.61	0.77	0.01
Leu	1.35	1.26	1.34	1.82	2.22	0.04
Lys	1.14	1.07	1.08	0.56	0.65	0.02
Met	0.24	0.22	0.37	0.35	0.35	0.01
Phe	0.90	0.83	0.78	0.75	0.96	0.02
Thr	0.69	0.64	0.81	0.62	0.70	0.01
Trp	0.25	0.21	0.22	0.12	0.16	0.01
Val	0.86	0.80	0.99	0.78	0.94	0.01
Nonessential amino acids, %						
Ala	0.77	0.73	0.85	1.14	1.35	0.02
Asp	2.04	1.88	1.40	1.07	1.31	0.02
Cys	0.29	0.26	0.51	0.38	0.34	0.01
Glu	3.31	3.11	3.63	2.73	3.21	0.05
Gly	0.75	0.71	0.99	0.65	0.69	0.01
Pro	0.88	0.83	1.18	1.37	1.39	0.02
Ser	0.78	0.73	0.72	0.71	0.82	0.01
Tyr	0.58	0.54	0.48	0.56	0.69	0.02

1 SBM: solvent-extracted soybean meal, ESBM: extruded soybean meal, CM: solvent-extracted canola meal, DDGS: corn dried distillers grains with solubles, and HP-DDGS: high-protein corn dried distillers grains with solubles.

### Birds and housing

A total of 504 one-day-old male broiler chicks of Cobb500 strain were obtained from a commercial hatchery. The birds were weighed and distributed to 84 cages in electrically heated Petersime battery brooders (Petersime Incubator Co., Gettysburg, OH, USA) with 6 birds per cage based on body weight. After two weeks of feeding a commercial starter diet, each cage housing six birds was weighed for diet allotment (average cage BW with 6 birds: 2,412 ± 189 g). Room temperature (30°C) and lighting were controlled according to breed housing recommendations. Brooder temperature was maintained at 33 ± 1 °C for the first week and 31 ± 1 °C for the second week. Diets and fresh water were provided to the birds *ad libitum* during the entire period.

### Experimental design and procedure

The ten experimental diets consisted of five diets without protease and five diets with protease. These diets were assigned to 80 cages, with six birds per cage, following a completely randomized design. Each diet was allocated to eight cages. Additionally, the two N-free diets, either without or with protease, were assigned to four additional cages, with six birds per cage and two cages per N-free diet.

From day 0 to 14, all birds were fed an antibiotic- and coccidiostat-free commercial starter diet (Start & Grow, non-medicated crumbles, Purina Animal Nutrition, Arden Hills, MN, USA). After which birds were provided the assigned experimental diets from day 14 to 21. On day 21, all birds were euthanized via cervical dislocation. Ileal digesta samples were collected from the Meckel’s diverticulum to the ileal-ceca junction. The collected excreta and ileal digesta samples were pooled for each cage and stored frozen at –20°C for further analysis, respectively.

### Sample preparation and analyses

The pooled ileal digesta samples were freeze-dried. The test feedstuffs, experimental diets, and dried ileal digesta samples were ground finely to pass through a 0.75-mm screen using a centrifugal mill (model Zm200; Retsh GmbH, Haan, Germany). The test feedstuffs and diets were analyzed for dry matter (**DM**), CP, ether extract (**EE**), crude ash, crude fiber, AA, and titanium content. The ileal digesta samples were analyzed for AA and titanium contents.

The samples were analyzed for DM by oven drying at 135°C for 2 h (method 930.15), CP by a combustion procedure (method 990.03), EE (method 2003.06), and crude ash (method 942.05) as per AOAC (2006). The samples were analyzed for AA (method 982.30 E) and crude fiber (method 978.10) at the University of Missouri Experiment Station laboratories (Colombia, MO, USA) using the AOAC (2006). The concentration of TiO_2_ in the samples were determined by spectrophotometry (model Spectra MAX 190, Molecular Devices, Sunnyvale, CA) at 408 nm after ashing at 525°C for 10 h (Myers et al., 2004).

### Digestibility calculations

Apparent ileal digestibility (AID) of AA was calculated using the index method (Kong and Adeola, 2014):

% apparent nutrient digestibility = [1 – (N_f_/N_d_) × (M_d_/M_f_)] × 100, where N_f_ = nutrient concentration in excreta or ileal digesta (% DM); N_d_ = nutrient concentration in the diet (% DM); M_d_ = titanium concentration in diet (% DM); M_f_ = titanium concentration in excreta or ileal digesta (% DM).

SID of AA for diets was calculated from AID of AA corrected for basal endogenous losses of AA using the following equation (Kong and Adeola, 2014):

SID, % = AID + [(basal IAA_end_/AA_diet_) × 100, where IAA_end_ = basal endogenous loss of an AA in grams per kilogram of DM intake; AAdiet represent the AA concentrations (g/kg) in diet DM.

Basal endogenous losses of AA from birds fed a N-free diet without protease were used for the diets without protease and basal endogenous losses of AA from the birds fed a N-free diet with protease were used for the diets with protease.

### Statistical analysis

The UNIVARIATE procedure of SAS (SAS Inst. Inc., Cary, NC, USA) was used to confirm the homogeneity of variance and to analyze for outliers. Data were subjected to analysis of variance using the MIXED procedure of SAS for a completely randomized design with cage as an experimental unit. Protein ingredient and protease supplementation were considered as main factors, and their interactions were also determined. Because of a significant interaction between ingredient and protease and differences in ingredient specific nutrient composition (see discussion for more details), a student’s t-test was used to evaluate the effect of protease supplementation in each protein ingredient (Supplementary Tables 1 and 2). Treatment means were separated by the probability of difference using Tukey’s adjustment where the main effects were observed. To test the hypotheses, *P* ≤ 0.05 was considered significant. If pertinent, tendency (0.05 < *P* ≤ 0.10) was also reported.

## Results

All birds were deemed healthy throughout the 2 to 3 weeks of the experimental diet based on the lack of abnormal behavior and mortality.

The CP content of HP-DDGS was the highest, whereas the CP content of DDGS was the lowest (Table 2). Leucine, Lys, and Arg were the most abundant essential AA in SBM, ESBM, and CM, whereas Met and Trp were the least abundant essential AA. Leucine was the most abundant essential AA in DDGS and HP-DDGS, whereas Met, Trp, and His were the least abundant essential AA in DDGS and Trp was the least abundant essential AA in HP-DGGS. The analyzed CP contents in the diets ranged from 16.2 % to 17.5 % (Table 3). The CM and DDGS diets had higher fiber contents than other diets.

An interaction between ingredient and protease was not detected in the final body weight of the birds at the end of the collection period (Table 4). There were interactions (*P* < 0.05) between ingredient and protease for AID of all analyzed essential AA (Table 5) and non-essential AA (Table 6) except Arg, Lys, and Met and there were interactions (*P* < 0.05) between ingredient and protease for SID of all analyzed essential AA (Table 7) and non-essential AA (Table 8) except Arg, Lys, Met, and Trp. The interactive effects between protein ingredient and protease supplementation showed that the AID of His, Ile, Leu, Phe, Thr, Val, and nonessential AA in the SBM (except for Glu) and HP-DDGS diets were improved with protease (*P* < 0.05), whereas the broilers fed the ESBM, CM, and DDGS diets had no improvement on the AID for these AA when diets were supplemented with protease (Table 5, Table 6). In addition, the SID of His, Ile, Leu, Phe, Thr, Val and nonessential AA in the SBM (except for Glu) and HP-DDGS diets were improved with protease supplementation (*P* < 0.05), whereas the broilers fed ESBM, CM and DDGS diets supplemented with protease had improvement in SID of these AA, showing a similar trend with AID (Table 7, Table 8).

**Table 4:** Bird body weight during the collection period from d 14 to 21.

Ingredient^1^	Protease^2^	Initial BW, g/bird	Final BW, g/bird
SBM	–	392	582
SBM	+	411	627
ESBM	–	410	605
ESBM	+	402	578
CM	–	410	710
CM	+	409	709
DDGS	–	388	511
DDGS	+	398	528
HP-DDGS	–	403	514
HP-DDGS	+	396	506
Pooled SEM		31.2	83.6
Main effects			
SBM		401	604^b^
ESBM		406	592^b^
CM		410	709^a^
DDGS		393	520^c^
HP-DDGS		400	510^c^
*P* – value		0.658	<.001
	–	400	585
	+	404	590
	*P* – value	0.673	0.591
Interactions			
*P* – value		0.736	0.168

^abc^Values with different superscripts within the columns are different (*P* < 0.05).

1 SBM: solvent-extracted soybean meal, ESBM: extruded soybean meal, CM: solvent-extracted canola meal, DDGS: corn dried distillers grains with solubles, and HP-DDGS: high-protein corn dried distillers grains with solubles.

2 Protease supplementation at 0 or 250 mg/kg.

**Table 5:** The interaction and main effects of protein ingredient or protease on AID of essential AA in broilers.

Ingredient^1^	Protease^2^	Arg	His	Ile	Leu	Lys	Met	Phe	Thr	Trp	Val
SBM	–	94.6	92.1^b^	90.1^b^	89.8^b^	91.7	91.4	91.1^b^	84.8^b^	93.1^b^	89.0^b^
SBM	+	96.0	94.1^a^	92.6^a^	92.5^a^	94.1	93.9	93.4^a^	88.8^a^	94.9^a^	91.3^a^
ESBM	–	96.2	93.4^ab^	92.1^a^	92.4^a^	93.8	93.0	93.6^a^	87.7^a^	94.0^ab^	91.4^a^
ESBM	+	96.9	94.4^a^	92.9^a^	93.0^a^	94.5	93.9	94.2^a^	88.3^a^	94.9^a^	92.1^a^
CM	–	91.4	89.3^c^	84.6^c^	87.1^c^	83.8	90.4	87.5^c^	79.6^c^	92.8^b^	83.9^c^
CM	+	91.6	89.3^c^	85.1^c^	87.1^c^	83.9	90.7	87.5^c^	79.3^c^	93.6^b^	84.4^c^
DDGS	–	85.4	82.8^d^	81.1^d^	88.7^bc^	73.0	86.2	85.1^d^	71.8^d^	81.1^d^	80.8^d^
DDGS	+	85.6	82.5^d^	80.9^d^	88.9^bc^	73.1	87.0	85.1^d^	72.1^d^	80.7^d^	80.5^d^
HP-DDGS	–	78.9	71.5^f^	71.0^f^	77.4^e^	69.1	76.3	75.6^f^	66.2^e^	81.5^d^	70.5^f^
HP-DDGS	+	80.7	76.4^e^	75.4^e^	82.0^d^	72.5	80.6	80.1^e^	70.4^d^	84.6^c^	75.1^e^
Pooled SEM		0.89	0.80	0.83	0.86	0.76	0.68	0.68	0.71	0.79	0.65
Main effects											
SBM		95.3^a^	93.1	91.3	91.2	92.9^a^	92.7^a^	92.3	86.8	94.0	90.4
ESBM		96.5^a^	93.9	92.5	92.7	94.1^a^	93.5^a^	93.9	88.0	94.5	91.7
CM		91.5^b^	89.3	84.9	87.1	83.8^b^	90.5^b^	87.5	79.5	93.2	84.2
DDGS		85.5^c^	82.7	81.0	88.8	73.1^c^	86.6^c^	85.1	72.0	80.9	80.6
HP-DDGS		79.8^d^	73.9	73.2	79.7	70.8^d^	78.5^d^	77.8	68.3	83.0	72.8
*P* – value		<.001	<.001	<.001	<.001	<.001	<.001	<.001	<.001	<.001	<.001
	–	89.3	85.8	83.8	87.1	82.3	87.5	86.6	78.0	88.5	83.1
	+	90.2	87.3	85.2	88.7	83.6	89.2	88.1	79.6	89.5	84.6
	*P* – value	0.175	0.001	0.004	<.001	0.016	0.001	0.001	0.006	0.011	0.004
Interactions											
*P* – value		0.894	0.002	0.009	0.003	0.224	0.105	0.004	0.008	0.053	0.005

^abcdef^Values with different superscripts within the columns are different (*P* < 0.05).

1 SBM: solvent-extracted soybean meal, ESBM: extruded soybean meal, CM: solvent-extracted canola meal, DDGS: corn dried distillers grains with solubles, and HP-DDGS: high-protein corn dried distillers grains with solubles.

2 Protease supplementation at 0 or 250 mg/kg.

**Table 6:** The interaction and main effects of protein ingredient or protease on AID of non-essential AA in broilers.

Ingredient^1^	Protease^2^	Ala	Asp	Cys	Glu	Gly	Pro	Ser	Tyr
SBM	–	89.3^b^	90.0^b^	83.7^b^	93.8^b^	88.0^b^	90.0^b^	89.0^b^	90.6^b^
SBM	+	92.1^a^	92.5^a^	87.8^a^	95.4^ab^	91.0^a^	92.3^a^	91.8^a^	93.1^a^
ESBM	–	91.5^a^	92.9^a^	86.6^a^	95.6^a^	89.7^ab^	91.6^ab^	91.0^a^	92.6^a^
ESBM	+	92.4^a^	93.3^a^	87.0^a^	96.2^a^	90.3^a^	92.3^a^	91.7^a^	93.0^a^
CM	–	86.7^c^	82.8^c^	82.2^b^	91.9^c^	84.9^c^	82.0^d^	82.0^c^	84.0^c^
CM	+	86.6^c^	82.5^c^	82.3^bc^	91.8^c^	85.0^c^	82.6^de^	82.9^c^	83.9^c^
DDGS	–	87.0^c^	74.8^d^	77.9^d^	87.7^d^	75.4^d^	85.5^c^	80.4^c^	85.5^c^
DDGS	+	87.2^c^	74.9^d^	78.7^cd^	87.9^d^	75.3^d^	85.4^c^	81.0^c^	85.7^c^
HP-DDGS	–	76.7^e^	67.1^f^	66.4^f^	77.0^f^	68.0^f^	74.5^f^	72.9^e^	77.9^e^
HP-DDGS	+	81.0^d^	71.5^e^	71.2^e^	81.4^e^	72.1^e^	79.4^e^	77.4^d^	82.0^d^
Pooled SEM		0.65	0.71	1.35	0.64	0.98	0.93	0.79	0.81
Main effects									
SBM		90.7	91.3	85.8	94.6	89.5	91.2	90.4	91.8
ESBM		91.9	93.1	86.8	95.9	90.0	92.0	91.3	92.8
CM		86.6	82.7	82.3	91.8	84.7	82.4	82.5	84.0
DDGS		87.1	74.9	78.3	87.8	75.3	85.4	80.7	85.6
HP-DDGS		78.9	69.3	68.8	79.2	70.0	76.9	75.2	79.9
*P* – value		<.001	<.001	<.001	<.001	<.001	<.001	<.001	<.001
	–	86.2	81.5	79.4	89.2	81.2	84.7	83.1	86.1
	+	87.9	82.9	81.2	90.5	82.6	86.1	84.5	87.5
	*P* – value	<.001	0.007	0.004	0.002	0.006	0.003	0.003	0.001
Interactions									
*P* – value		0.006	0.020	0.015	0.007	0.020	0.001	0.004	0.006

^abcdef^Values with different superscripts within the columns are different (*P* < 0.05).

1 SBM: solvent-extracted soybean meal, ESBM: extruded soybean meal, CM: solvent-extracted canola meal, DDGS: corn dried distillers grains with solubles, and HP-DDGS: high-protein corn dried distillers grains with solubles.

2 Protease supplementation at 0 or 250 mg/kg.

**Table 7:** The interaction and main effects of protein ingredient or protease on SID of essential AA in broilers.

Ingredient^1^	Protease^2^	Arg	His	Ile	Leu	Lys	Met	Phe	Thr	Trp	Val
SBM	–	95.3	92.1^b^	90.6^b^	90.8^b^	92.6	93.6	92.2^b^	85.2^b^	95.7	89.5^b^
SBM	+	96.8	94.1^a^	93.2^a^	93.6^a^	95.3	96.6	94.7^a^	89.3^a^	98.0	92.4^a^
ESBM	–	97.2	93.4^ab^	92.8^a^	93.7^a^	95.0	95.8	95.2^a^	88.2^a^	97.4	92.0^a^
ESBM	+	98.4	94.4^a^	93.9^a^	94.8^a^	96.4	98.2	96.5^a^	89.0^a^	99.9	92.9^a^
CM	–	91.9	89.3^c^	85.0^c^	87.9^c^	84.3	91.7	88.5^cd^	79.9^c^	95.8	84.2^c^
CM	+	92.3	89.3^c^	85.6^c^	88.2^c^	84.6	92.5	88.7^c^	79.7^c^	96.5	84.5^c^
DDGS	–	86.0	82.8^d^	81.6^d^	89.6^bc^	73.8	87.5	86.2^e^	72.2^d^	83.8	81.2^d^
DDGS	+	86.3	82.5^d^	81.5^d^	89.9^bc^	74.1	88.5	86.4^de^	72.5^d^	83.9	80.9^d^
HP-DDGS	–	79.2	71.5^f^	71.2^f^	77.6^e^	69.5	76.8	75.9^g^	66.4^e^	82.9	70.7^f^
HP-DDGS	+	81.0	76.4^e^	75.7^e^	82.3^d^	73.0	81.3	80.6^f^	70.6^d^	86.3	75.3^e^
Pooled SEM		1.89	1.67	1.78	1.61	1.75	1.34	1.51	1.95	1.53	1.67
Main effects											
SBM		96.0^a^	93.1	91.9	92.2	94.0^b^	95.1^b^	93.5	87.2	96.8^b^	90.9
ESBM		97.8^a^	93.9	93.3	94.3	95.7^a^	97.0^a^	95.8	88.6	98.7^a^	92.4
CM		92.1^b^	89.3	85.3	88.0	84.4^c^	92.1^c^	88.6	79.8	96.2^b^	84.3
DDGS		86.2^c^	82.7	81.5	90.0	73.9^d^	88.0^d^	86.3	72.4	83.8^c^	81.1
HP-DDGS		80.1^d^	73.9	73.4	80.0	71.2^e^	79.0^e^	78.2	68.5	84.6^c^	73.0
*P* – value		<.001	<.001	<.001	<.001	<.001	<.001	<.001	<.001	<.001	<.001
	–	89.9	85.8	84.2	87.9	83.1	89.1	87.6	78.4	91.1	83.5
	+	91.0	87.3	85.8	89.8	84.7	91.4	89.4	80.0	92.9	85.1
	*P* – value	0.103	0.001	0.002	<.001	0.006	<.001	<.001	0.004	<.001	0.002
Interactions											
*P* – value		0.921	0.002	0.011	0.008	0.286	0.275	0.009	0.010	0.165	0.006

^abcdefg^Values with different superscripts within the columns are different (*P* < 0.05).

1 SBM: solvent-extracted soybean meal, ESBM: extruded soybean meal, CM: solvent-extracted canola meal, DDGS: corn dried distillers grains with solubles, and HP-DDGS: high-protein corn dried distillers grains with solubles.

2 Protease supplementation at 0 or 250 mg/kg.

**Table 8:** The interaction and main effects of protein ingredient or protease on SID of non-essential AA in broilers.

Ingredient^1^	Protease^2^	Ala	Asp	Cys	Glu	Gly	Pro	Ser	Tyr
SBM	–	90.4^b^	90.5^b^	84.7^b^	94.2^b^	88.5^b^	91.0b	89.5^b^	92.2^b^
SBM	+	93.4^a^	93.0^a^	88.9^a^	95.9^ab^	91.6^a^	93.5^a^	92.4^a^	95.0^a^
ESBM	–	92.9^a^	93.5^a^	87.8^a^	96.2^a^	90.3^ab^	92.8^ab^	91.6^a^	94.6^a^
ESBM	+	94.3^a^	94.1^a^	88.6^a^	97.1^a^	91.1^a^	94.0^a^	92.5^a^	95.8^a^
CM	–	87.5^c^	83.2^c^	82.8^bc^	92.2^c^	85.3^c^	82.5^d^	82.3^c^	85.3^d^
CM	+	87.7^c^	83.0^c^	83.0^cd^	92.2^c^	85.5^c^	82.8^de^	82.4^c^	85.6^cd^
DDGS	–	87.8^c^	75.3^d^	78.7^e^	88.0^d^	75.8^d^	86.1^c^	80.9^c^	87.0^cd^
DDGS	+	88.2^c^	75.4^d^	79.6^de^	88.3^d^	75.7^d^	86.1^c^	81.6^c^	87.5^c^
HP-DDGS	–	77.0^e^	67.3^f^	66.8^g^	77.1^f^	68.2^f^	74.7^f^	73.1^e^	78.4^f^
HP-DDGS	+	81.4^d^	71.7^e^	71.7^f^	81.5^e^	72.3^e^	79.7^e^	77.7^d^	82.7^e^
Pooled SEM		1.57	1.93	2.50	1.39	1.72	1.82	1.83	1.74
Main effects									
SBM		91.9	91.7	86.8	95.1	90.0	92.3	90.9	93.6
ESBM		95.6	93.8	88.2	96.6	90.7	93.4	92.1	95.2
CM		87.6	83.1	82.9	92.2	85.4	82.6	82.3	85.4
DDGS		88.0	75.4	79.1	88.2	75.8	86.1	81.2	87.2
HP-DDGS		79.2	69.5	69.2	79.3	70.2	77.2	75.4	80.6
*P* – value		<.001	<.001	<.001	<.001	<.001	<.001	<.001	<.001
	–	87.1	82.0	80.1	89.5	81.6	85.4	83.5	87.5
	+	89.0	83.4	82.2	91.0	83.1	86.9	85.1	89.3
	*P* – value	<.001	0.004	0.002	0.001	0.004	0.001	0.001	<.001
Interactions									
*P* – value		0.012	0.026	0.020	0.010	0.023	0.001	0.005	0.021

^abcdefg^Values with different superscripts within the columns are different (*P* < 0.05).

1 SBM: solvent-extracted soybean meal, ESBM: extruded soybean meal, CM: solvent-extracted canola meal, DDGS: corn dried distillers grains with solubles, and HP-DDGS: high-protein corn dried distillers grains with solubles.

2 Protease supplementation at 0 or 250 mg/kg.

The SBM and ESBM diets showed greater (*P* < 0.05) AID of AA than the other diets. Between SBM and ESBM diets, the AID of Leu, Phe, Asp, and Glu was greater (*P* < 0.05) in the ESBM diet than in the SBM diet. However, the SID of Ile, Leu, Lys, Met, Phe, Trp, Val, Ala, Asp, Glu, and Tyr was greater (*P* < 0.05) in the ESBM diet than in the SBM diet. The AID of essential AA (except for Leu) in the CM diet was greater (*P* < 0.05) than in the DDGS and HP-DDGS diets, with the DDGS diet showed greater AID of AA than the HP-DDGS diet. Furthermore, amongst CM, DDGS, and HP-DDGS diets, the SID of essential AA (except for Leu and Trp) as wells as Ala, Pro, Ser, and Tyr was greater (*P* < 0.05) in the CM diet, followed by the DDGS diet, and lowest in the HP-DDGS diet.

## Discussion

It is well documented that protease supplementation in monogastric animal diets can improve the ileal digestibility of AA (Cowieson and Roos, 2014; Lee et al., 2018). Protease can hydrolyze protein-bound starches in feed ingredients, releasing additional protein and energy substrates, and degrade protein anti-nutritional factors (e.g. trypsin inhibitor), which can otherwise reduce protein digestibility in poultry (Nu et al., 2018; McCafferty et al., 2022). Some studies reported that protease supplementation may resulted in a greater magnitude of improvement in protein or AA digestibility in low-protein diets, possibly due to reduced endogenous enzyme secretion or improved nutrient accessibility (Ravindran, 2013; Jabbar et al., 2021). Freitas et al. (2011) indicated that broilers fed a corn-SBM-based diet with higher energy and protein contents showed a significant improvement in ileal digestibility of CP with protease supplementation compared with a corn-SBM-based diet with lower energy and protein contents. However, this response may also be influenced by the lower substrate availability in such low-protein diets, which can limit the absolute effect of exogenous protease (Law et al., 2015).

In the present study, the diets were formulated such that each protein ingredient contributed 16.6 % to 16.8 % diet CP, thus the analyzed CP content of each experimental diet ranged from 16.2 % to 17.5 %. To evaluate the efficacy of protease supplementation on protein and AA digestibility in individual protein ingredients, experimental diets were formulated using various protein ingredients, including SBM, ESBM, CM, DDGS, and HP-DDGS, each as the sole protein source. The interaction response between protease supplementation and protein source varied, with improvements averaging 4.3, 2.7, and 1.2 percentage units in HP-DDGS, SBM, and ESBM, respectively, while no significant impact was observed in CM and DDGS. The diet formulation and experimental design aimed to assess the impact of dietary protease on AA digestibility in these protein sources when protein substrate (i.e., CP content in the diet) was consistent across all test diets. However, other nutrients (e.g., simple and complex carbohydrates and fat) were not equal across diets, which has previously been reported to influence the observed response in AA digestibility in pigs (Cowieson and Roos, 2014). Therefore, it can be speculated that the observed differential protease effect among the tested ingredients in the present study was more strongly influenced by the fiber content or the protein-fiber matrix of the ingredient rather than the CP level of the ingredient or diet. For example, the SBM used in the present study contained lower fiber content than CM and cDDGS, while HP-DDGS also had relatively lower fiber content than CM and cDDGS. Compared to cDDGS, HP-DDGS undergoes additional processing technologies, such as kernel fractionation or dry fractionation process, resulting in a higher protein content and a lower proportion of fiber and other non-essential components (Applegate et al., 2009). In the diet formulation of the current study, the lower inclusion levels of SBM (35 %) and HP-DDGS (33 %) in the diets, compared to CM (44 %) and DDGS (55 %), were due to their higher CP content. This resulted in differing crude fiber contents among the diets (Table 2: 3.64 % and 6.68 % for SBM and HP-DDGS, respectively; 14.53 % and 8.60 % for CM and DDGS, respectively), which may have reduced the efficacy of protease hydrolysis. Vivares et al., (2025) indicated that a higher proportion of soluble dietary fiber is negatively correlated with DM and CP digestibility in broilers. This is attributed to the ingredient-specific characteristics of dietary fiber, such as polymer composition and physicochemical properties like water holding capacity and extract viscosity.

The composition and structure of the protein matrix, which is linked with carbohydrates and other nutrients could be a major factor influencing protease efficacy in feed ingredients (Acamovic, 2001). Corn DDGS contains protein-polyphenol-carbohydrate complexes formed during the heat treatment in the ethanol production process (Rizzi, 2003; Le Bourvellec and Renard, 2012). As a result, proteins encapsulated within the fiber matrix may not easily be accessed by exogenous enzymes due to the complex carbohydrate structure, which features a backbone of β-(1-4) linked xylose units and side chains of arabinose, glucuronic acid, acetyl acid, and phenolic acid (Mendis and Simsek, 2014). Similarly, conventional CM, a byproduct of *Brassica* oilseed produced by solvent-extraction, contains protein-carbohydrate complexes. These complexes result from heat-damaged protein and the formation of advanced Maillard reaction products, which resemble neutral detergent fiber and lignin (Bell, 1993). These complexes can lead to reduced nutrient digestibility in poultry (Woyengo and Nyachoti, 2011). Furthermore, it should be noted that canola seeds are approximately 4 to 5 times smaller than soybeans, implying that the residual nutrients and fiber in CM would be more compact and condensed compared to those in SBM. This difference in physiochemical structure can result in more highly lignified fiber in CM than in SBM (Khajali and Slominski, 2012), and consequently, less fermentable fiber in CM (Woyengo et al., 2016).

Similar to the observation in the present study, Barekatain et al. (2013) reported that protease supplementation (200 mg protease/ kg of diet) in broiler diets containing sorghum DDGS at 0 to 30 % did not affect the AID of AA. In contrast, Siegert et al. (2019) found that protease supplementation (1,600 mg protease/kg of diet) in diets containing 35 % SBM, 20 % SBM-20 % RSM, and 20 % SBM-20 % sunflower meal improved the ileal digestibility of CP and AA, except for Cys. However, there was an interaction between protease and protein source such that dietary protease improved the ileal digestibility of Cys for the 35 % SBM diet and the 20 % SBM-20 % sunflower meal diet, whereas it did not affect ileal digestibility of Cys for 20 % SBM-20 % RSM diet. The authors argued that the controversial results regarding the effect of protease supplementation on AA digestibility might be due to the ingredient composition of the diet, as it directly influences the availability of protein substrates for enzymes to act upon. The ingredient composition may also modify other conditions in the digestive tract that affect enzyme activity. Liu et al. (2024) reported that protease supplementations (50 mg protease/kg of diet and 30,000 protease units/kg of diet) improved the AID coefficients for most AA in SBM (except for His, Trp, and Gly) and RSM (except for Met and Cys) in broilers. It should be noted that the CP content of SBM diets were greater than those of RSM diets by 1.6 percentage unit in the study of Liu et al. (2024). Erdaw et al. (2017a) reported that protease supplementation, ranging from 0.1 g protease/kg of diet (7,500 protease unit/kg of diet) to 0.3 g protease/kg of diet (22,500 protease unit/kg of diet), did not improve the ileal AA digestibility of broilers fed the diets containing 0 to 20 % of raw full-fat soybeans. Thus, the improvements in AA digestibility for SBM and HP-DDGS with protease supplementation could partly be attributed to the higher and more digestible protein content in these protein sources, which may require more protein hydrolysis than what endogenous enzyme activity can provide. Furthermore, the lower fiber content in SBM and HP-DDGS compared CM and DDGS may have enhanced protease accessibility and efficacy, contributing to the observed improvements. Therefore, both protein quality and fiber composition should be considered when interpreting the response to exogenous enzyme supplementation.

Notwithstanding, it is possible that the protease dosage, endogenous enzyme secretion activity, or other factors contributed to the responses observed in the current study. Therefore, the effects were not likely solely attributable to the chemical composition or physio-chemical matrix characteristics of the protein ingredients. Cowieson and Roos (2014) discussed the findings of Huang et al. (2005), noting that the response to protease effects on AA digestibility was greater in wheat-canola-based diet than in corn-SBM-based diet. Liu et al. (2024) observed a greater response to dietary protease on the AID of AA in RSM than in SBM. They inferred that this difference in protease response was due to variations in inherent AA digestibility, which could be influenced by factors such as age (Huang et al., 2005), supplemental AA and AA density (Fan et al., 1994; Selle et al., 2007), ingredient type and quality (Lemme et al., 2004; Liu et al., 2013), and antinutritional factors (Cowieson et al., 2004; Clarke and Wiseman, 2005). Cowieson et al. (2020) indicated that bird body weight and feed intake during the experimental period are important, which is known to significantly influence digestibility values (Fan et al., 1994). In the present study, final body weight was not affected by the interaction between ingredient and protease and by the main effect of protease. However, feed intake was not measured during the collection period, which limits our ability to determine whether the observed improvements in AA digestibility were genuinely a result of protease supplementation or confounded by changes in feed (nutrient) intake, digesta flow, or digestive capacity. Given the limitations of the current study, the results should be interpreted with caution when practical applications.

In the present study, protease did influence the AA digestibility for SBM whereas it did not influence the AA digestibility for ESBM. The reason for this difference between SBM and ESBM is not clear and could partly have been attributed to the proportion of the protein, fat, and fiber contents in those ingredients. Cowieson and Roos (2014) suggested that the efficacy of exogenous protease could be mediated through a reduction in mucoprotein loss from the intestine; it might be associated with a decrease in the mucin secretion or an increase in the autolytic recovery of mucin. Ileal endogenous AA losses, including digestive secretion, mucoproteins, and sloughed intestinal epithelial cells (Moughan and Schutter, 1991; Ravindran and Bryden, 1999), are related to the composition of the dietary feedstuff, including fiber types, antinutritional factors, and dietary protein level (Stein et al., 2007; Kong and Adeola, 2014). Erdaw et al. (2017b) reported that increasing the level of hammer-milled full-fat soybeans, replacing conventional SBM from 0 to 25 %, increased basal endogenous N loss, which resulted in a decrease in both AID and SID of AA. They also found that protease supplementation (15,000 protease unit/kg of diet) had no effect on the AID and SID of AA (except for Lys) in the full-fat soybeans diets. Furthermore, the ESBM diet had a higher trypsin inhibitor activity than the SBM diet, as reported in our previous study (Atoo et al., 2025; ESBM: 6.18 TIU/mg, SBM: 5.11 TIU/mg), which may have reduced the effectiveness of both endogenous and exogenous protease enzymes in broilers fed the ESBM diet. Interestingly, the observed difference in the interactive effects of dietary protease and protein ingredient on the AID and SID of Trp is because dietary protease did not affect the AID of Trp for ESBM but improved the SID of Trp for ESBM. Cowieson and Roos (2014) indicated that the magnitude of dietary protease effects was notably lower for the AID of Trp (the second lowest after Glu), which could partly be attributed to the higher inherent AA digestibility in broilers.

While not a specific objective of the current study it is of interest to note that the basal ileal endogenous AA loss in broilers fed the N-free diet with protease was numerically greater than in broilers fed the N-free diet without protease by 19.6 % (Supplementary Table 1). However, this numerical difference in basal endogenous AA loss with protease supplementation resulted in only a very small difference (less than 1 %) in the SID of AA values (data not presented) when the N-free without protease was used for all test diets. However, it should be noted that the feedstuffs used in the present study were high-CP ingredients containing fiber and anti-nutrients, which may have influenced both basal endogenous N loss and specific endogenous AA loss (Ravindran, 2016). Therefore, future digestibility studies with protease should consider both the CP content of the evaluated feedstuffs and the potential effect of dietary protease on both basal and specific endogenous AA loss in broilers.

## Conclusion

In conclusion, protease supplementation in broiler diets could improve the SID of AA for the SBM, ESBM, and HP-DDGS. The lack of difference in SID of AA for CM and DDGS with protease inclusion could partly have been due to the relatively higher fiber and lower protein contents and their protein-fiber matrix complexity than other soybean byproducts and HP-DDGS. This highlights the importance of considering ingredient-specific characteristics, such as fiber content and protein quality, when formulating broiler diets with exogenous enzymes. Our findings suggest that protease supplementation is particularly beneficial in diets containing high-protein, low-fiber byproducts like SBM and HP-DDGS, whereas additional enzyme strategies (e.g., fiber-degrading enzymes) may be needed to improve AA digestibility in fibrous protein sources such as CM and DDGS.

## Disclosures

The authors declare the following financial interests/personal relationships which may be considered as potential competing interests:

Crystal Levesque reports financial support was provided by CBS Bio Platforms Inc. If there are other authors, they declare that they have no known competing financial interests or personal relationships that could have appeared to influence the work reported in this paper.

## Appendix Supplementary materials

Image, application 1

## Footnotes

Supplementary material associated with this article can be found, in the online version, at doi:10.1016/j.psj.2025.105605.
