# Supplementary material for: Effects of protease supplementation on the ileal digestibility of amino acids for protein ingredients in broiler chickens
Source: Poult Sci. 2025 Jul 25;104(10):105605. doi: 10.1016/j.psj.2025.105605 (PMC12329081; doi:10.1016/j.psj.2025.105605)
Supplement: Supplementary file 1 [file mmc1.docx]

**Supplementary Table 1**. The differential response of dietary protease in protein ingredients on AID of AA in broilers

| Ingredient^1^ | SBM | | SEM^3^ | *P^4^* | ESBM | | SEM | *P* | CM | | SEM | *P* | DDGS | | SEM | *P* | HP-DDGS | | SEM | *P* |
| --- | --- | --- | --- | --- | --- | --- | --- | --- | --- | --- | --- | --- | --- | --- | --- | --- | --- | --- | --- | --- |
| Protease^2^ | – | + |  |  | – | + |  |  | – | + |  |  | – | + |  |  | – | + |  |  |
| Essential amino acids, % | | | |  |  |  |  |  |  |  |  |  |  |  |  |  |  |  |  |  |
| Arg | 94.6 | 96.0 | 0.21 | ** | 96.2 | 96.9 | 0.28 | NS | 91.4 | 91.6 | 0.28 | NS | 85.4 | 85.6 | 0.46 | NS | 78.9 | 80.7 | 2.16 | NS |
| His | 92.1 | 94.1 | 0.26 | ** | 93.4 | 94.4 | 0.33 | † | 89.3 | 89.3 | 0.24 | NS | 82.8 | 82.5 | 0.34 | NS | 71.5 | 76.4 | 1.37 | * |
| Ile | 90.1 | 92.6 | 0.30 | ** | 92.1 | 92.9 | 0.37 | NS | 84.6 | 85.1 | 0.37 | NS | 81.1 | 80.9 | 0.31 | NS | 71.0 | 75.4 | 1.54 | † |
| Leu | 89.8 | 92.5 | 0.35 | ** | 92.4 | 93.0 | 0.37 | NS | 87.1 | 87.1 | 0.26 | NS | 88.7 | 88.9 | 0.20 | NS | 77.4 | 82.0 | 1.36 | * |
| Lys | 91.7 | 94.1 | 0.37 | ** | 93.8 | 94.5 | 0.47 | NS | 83.8 | 83.9 | 0.43 | NS | 73.0 | 73.1 | 0.80 | NS | 69.1 | 72.5 | 1.63 | NS |
| Met | 91.4 | 93.9 | 0.53 | ** | 93.0 | 93.9 | 0.66 | NS | 90.4 | 90.7 | 0.35 | NS | 86.2 | 87.0 | 0.43 | NS | 76.3 | 80.6 | 1.51 | † |
| Phe | 91.1 | 93.4 | 0.30 | ** | 93.6 | 94.2 | 0.33 | NS | 87.5 | 87.5 | 0.27 | NS | 85.1 | 85.1 | 0.25 | NS | 75.6 | 80.1 | 1.40 | * |
| Thr | 84.8 | 88.8 | 0.57 | ** | 87.7 | 88.3 | 0.59 | NS | 79.6 | 79.3 | 0.41 | NS | 71.8 | 72.1 | 0.54 | NS | 66.2 | 70.4 | 1.64 | † |
| Trp | 93.1 | 94.9 | 0.35 | ** | 94.0 | 94.9 | 0.47 | NS | 92.8 | 93.6 | 0.38 | NS | 81.1 | 80.7 | 0.76 | NS | 81.5 | 84.6 | 0.97 | * |
| Val | 89.0 | 91.8 | 0.37 | ** | 91.4 | 92.1 | 0.40 | NS | 83.9 | 84.4 | 0.33 | NS | 80.8 | 80.5 | 0.29 | NS | 70.5 | 75.1 | 1.56 | † |
| Nonessential amino acids, % | | | |  |  |  |  |  |  |  |  |  |  |  |  |  |  |  |  |  |
| Ala | 89.3 | 92.1 | 0.36 | ** | 91.5 | 92.4 | 0.42 | NS | 86.7 | 86.6 | 0.25 | NS | 87.0 | 87.2 | 0.22 | NS | 76.7 | 81.0 | 1.34 | * |
| Asp | 90.0 | 92.5 | 0.32 | ** | 92.9 | 93.3 | 0.32 | NS | 82.8 | 82.5 | 0.33 | NS | 74.8 | 74.9 | 0.49 | NS | 67.1 | 71.5 | 1.61 | * |
| Cys | 83.7 | 87.8 | 0.62 | ** | 86.6 | 87.0 | 0.52 | NS | 82.2 | 82.3 | 0.43 | NS | 77.9 | 78.7 | 0.41 | NS | 66.4 | 71.2 | 1.90 | † |
| Glu | 93.8 | 95.4 | 0.20 | ** | 95.6 | 96.2 | 0.24 | NS | 91.9 | 91.8 | 0.15 | NS | 87.7 | 87.9 | 0.24 | NS | 77.0 | 81.4 | 1.40 | * |
| Gly | 88.0 | 91.0 | 0.41 | ** | 89.7 | 90.3 | 0.40 | NS | 84.9 | 85.0 | 0.18 | NS | 75.4 | 75.3 | 0.39 | NS | 68.0 | 72.1 | 1.63 | NS |
| Pro | 90.0 | 92.3 | 0.36 | ** | 91.6 | 92.3 | 0.39 | NS | 82.0 | 82.6 | 0.41 | NS | 85.5 | 85.4 | 0.21 | NS | 74.5 | 79.4 | 1.35 | * |
| Ser | 89.0 | 91.8 | 0.42 | ** | 91.0 | 91.7 | 0.43 | NS | 82.0 | 82.9 | 0.37 | NS | 804 | 81.0 | 0.46 | NS | 72.9 | 77.4 | 1.43 | * |
| Tyr | 90.6 | 93.1 | 0.41 | ** | 92.6 | 93.0 | 0.35 | NS | 84.0 | 83.9 | 0.37 | NS | 85.5 | 85.7 | 0.35 | NS | 77.9 | 82.0 | 1.23 | * |

^1^SBM: solvent-extracted soybean meal, ESBM: extruded soybean meal, CM: solvent-extracted canola meal, DDGS: corn dried distillers grains with solubles, and HP-DDGS: high-protein corn dried distillers grains with solubles.

^2^Protease supplementation at 0 or 250 mg/kg.

^3^Standard error of the mean.

^4^Probability values were indicated as follows: **P* ≤ 0.05, ***P* ≤ 0.01, †*P* ≤ 0.10, and “NS” *P* > 0.10.

**Supplementary Table 2**. The differential response of dietary protease in protein ingredients on SID of AA in broilers.

| Ingredient^1^ | SBM | | SEM^3^ | *P^4^* | ESBM | | SEM | *P* | CM | | SEM | *P* | DDGS | | SEM | *P* | HP-DDGS | | SEM | *P* |
| --- | --- | --- | --- | --- | --- | --- | --- | --- | --- | --- | --- | --- | --- | --- | --- | --- | --- | --- | --- | --- |
| Protease^2^ | – | + |  |  | – | + |  |  | – | + |  |  | – | + |  |  | – | + |  |  |
| Essential amino acids, % | | | |  |  |  |  |  |  |  |  |  |  |  |  |  |  |  |  |  |
| Arg | 95.3 | 96.8 | 0.23 | ** | 97.2 | 98.4 | 0.34 | * | 91.9 | 92.3 | 0.30 | NS | 86.0 | 86.3 | 0.46 | NS | 79.2 | 81.0 | 2.18 | NS |
| His | 92.1 | 94.1 | 0.27 | ** | 93.4 | 94.4 | 0.33 | † | 89.3 | 89.3 | 0.24 | NS | 82.8 | 82.5 | 0.34 | NS | 71.5 | 76.4 | 1.36 | * |
| Ile | 90.6 | 93.2 | 0.30 | ** | 92.8 | 93.9 | 0.38 | † | 85.0 | 85.6 | 0.38 | NS | 81.6 | 81.5 | 0.32 | NS | 71.2 | 75.7 | 1.54 | † |
| Leu | 90.8 | 93.6 | 0.37 | ** | 93.7 | 94.8 | 0.41 | † | 87.9 | 88.2 | 0.29 | NS | 89.6 | 89.9 | 0.23 | NS | 77.6 | 82.3 | 1.36 | * |
| Lys | 92.6 | 95.3 | 0.41 | ** | 95.0 | 96.4 | 0.53 | † | 84.3 | 84.6 | 0.44 | NS | 73.8 | 74.1 | 0.85 | NS | 69.5 | 73.0 | 1.63 | NS |
| Met | 93.6 | 96.6 | 0.70 | ** | 95.8 | 98.2 | 0.90 | † | 91.7 | 92.5 | 0.41 | NS | 87.5 | 88.5 | 0.48 | NS | 76.8 | 81.3 | 1.53 | † |
| Phe | 92.2 | 94.7 | 0.33 | ** | 95.2 | 96.5 | 0.38 | * | 88.5 | 88.7 | 0.30 | NS | 86.2 | 86.4 | 0.29 | NS | 75.9 | 80.6 | 1.40 | * |
| Thr | 85.2 | 89.3 | 0.58 | ** | 88.2 | 89.0 | 0.60 | NS | 79.9 | 79.7 | 0.40 | NS | 72.2 | 72.5 | 0.55 | NS | 66.4 | 70.6 | 1.64 | † |
| Trp | 95.7 | 98.0 | 0.46 | ** | 97.4 | 99.9 | 0.72 | * | 95.8 | 96.5 | 0.53 | NS | 83.8 | 83.9 | 0.90 | NS | 82.9 | 86.3 | 1.01 | * |
| Val | 89.5 | 92.4 | 0.38 | ** | 92.0 | 92.9 | 0.41 | NS | 84.2 | 84.5 | 0.33 | NS | 81.2 | 80.9 | 0.30 | NS | 70.7 | 75.3 | 1.56 | † |
| Nonessential amino acids, % | | | |  |  |  |  |  |  |  |  |  |  |  |  |  |  |  |  |  |
| Ala | 90.4 | 93.4 | 0.39 | ** | 92.9 | 94.3 | 0.44 | * | 87.5 | 87.7 | 0.28 | NS | 87.8 | 88.2 | 0.24 | NS | 77.0 | 81.4 | 1.34 | * |
| Asp | 90.5 | 93.0 | 0.33 | ** | 93.5 | 94.1 | 0.33 | NS | 83.2 | 83.0 | 0.33 | NS | 75.3 | 75.4 | 0.51 | NS | 67.3 | 71.7 | 1.61 | † |
| Cys | 84.7 | 88.9 | 0.62 | ** | 87.8 | 88.6 | 0.54 | NS | 82.8 | 83.0 | 0.43 | NS | 78.7 | 79.6 | 0.43 | NS | 66.8 | 71.7 | 1.90 | † |
| Glu | 94.2 | 95.9 | 0.21 | ** | 96.2 | 97.1 | 0.26 | * | 92.2 | 92.2 | 0.16 | NS | 88.0 | 88.3 | 0.26 | NS | 77.1 | 81.5 | 1.41 | * |
| Gly | 88.5 | 91.6 | 0.42 | ** | 90.3 | 91.1 | 0.41 | NS | 85.3 | 85.5 | 0.18 | NS | 75.8 | 75.7 | 0.40 | NS | 68.2 | 72.3 | 1.63 | NS |
| Pro | 91.0 | 93.5 | 0.37 | ** | 92.8 | 94.0 | 0.42 | † | 82.5 | 82.8 | 0.41 | NS | 86.1 | 86.1 | 0.22 | NS | 74.7 | 79.7 | 1.36 | * |
| Ser | 89.5 | 92.4 | 0.43 | ** | 91.6 | 92.5 | 0.44 | NS | 82.3 | 82.4 | 0.38 | NS | 80.9 | 81.6 | 0.48 | NS | 73.1 | 77.7 | 1.43 | * |
| Tyr | 92.2 | 95.0 | 0.47 | ** | 94.6 | 95.8 | 0.41 | † | 85.3 | 85.6 | 0.41 | NS | 87.0 | 87.5 | 0.41 | NS | 78.4 | 82.7 | 1.24 | * |

^1^SBM: solvent-extracted soybean meal, ESBM: extruded soybean meal, CM: solvent-extracted canola meal, DDGS: corn dried distillers grains with solubles, and HP-DDGS: high-protein corn dried distillers grains with solubles.

^2^Protease supplementation at 0 or 250 mg/kg.

^3^Standard error of the mean.

^4^Probability values were indicated as follows: **P* ≤ 0.05, ***P* ≤ 0.01, †*P* ≤ 0.10, and “NS” *P* > 0.10.
